# Supplementary material for: Generation of endogenous pH-sensitive EGF receptor and its application in high-throughput screening for proteins involved in clathrin-mediated endocytosis
Source: eLife. 2019 May 8;8:e46135. doi: 10.7554/eLife.46135 (PMC6533059; doi:10.7554/eLife.46135)
Supplement: Figure 4—source data 1. — The data are sorted by the extent of the inhibition of FAP-EGFR endocytosis (expressed as average value of the difference from median of controls (non-targeting siRNA); n = 3) from the lowest to highest values. [file elife-46135-fig4-data1.pdf]

		Difference from median of controls									
Well Name	Well #	1st	2nd	3rd	Average	Standard deviation	SSMD	GeneSymbol	GeneId	GeneAccession	GINumber
E01	49	-1.266	-1.134	-1.060	-1.153	0.104	-6.248	Dynamin-2			
F01	61	-1.246	-1.127	-1.033	-1.135	0.107	-6.012	Dynamin-2			
E12	60	-1.211	-1.111	-1.053	-1.125	0.080	-7.940	Dynamin-2			
F12	72	-1.221	-1.100	-1.040	-1.120	0.092	-6.854	Dynamin-2			
G12	84	-1.024	-0.691	-0.905	-0.873	0.169	-2.914	Clathrin heavy chain			
H12	96	-1.044	-0.586	-0.889	-0.840	0.233	-2.035	Clathrin heavy chain			
G01	73	-0.932	-0.604	-0.887	-0.807	0.178	-2.559	Clathrin heavy chain			
H01	85	-0.952	-0.553	-0.856	-0.787	0.208	-2.133	Clathrin heavy chain			
F09	69	-0.608	-0.474	-0.503	-0.528	0.070	-4.239	PPP2R2A	5520	NM_002717	32307111
D07	43	-0.382	-0.246	-0.348	-0.325	0.070	-2.606	PTPRH	5794	NM_002842	67190343
E08	56	-0.297	-0.334	-0.291	-0.307	0.023	-7.494	INPP4A	3631	NM_001566	4504704
E06	54	-0.356	-0.236	-0.313	-0.302	0.061	-2.793	PR48	28227	NM_199326	41349490
D09	45	-0.269	-0.269	-0.297	-0.278	0.016	-9.780	FLJ23751	92370	NM_152282	22748648
H07	91	-0.309	-0.141	-0.258	-0.236	0.087	-1.538	SET	6418	NM_003011	142361523
F05	65	-0.201	-0.195	-0.229	-0.208	0.018	-6.554	DKFZP566K0524	26095	NM_001042365	109138576
D05	41	-0.214	-0.158	-0.247	-0.206	0.045	-2.575	PTPN2	5771	NM_002828	18104978
F10	70	-0.196	-0.144	-0.200	-0.180	0.031	-3.253	PFKFB3	5209	NM_004566	42476167
E04	52	-0.173	-0.154	-0.194	-0.174	0.020	-5.002	EYA2	2139	NM_172110	26667229
G02	74	-0.166	-0.176	-0.158	-0.167	0.009	-10.001	PTPN11	5781	NM_002834	33356176
G09	81	-0.220	-0.104	-0.166	-0.163	0.058	-1.581	FLJ22405	64419	NM_022485	117938273
F07	67	-0.171	-0.158	-0.157	-0.162	0.008	-11.419	G6PC	2538	NM_000151	119393889
B07	19	-0.143	-0.156	-0.173	-0.157	0.015	-5.907	MTMR4	9110	NM_004687	31377795
F04	64	-0.167	-0.155	-0.142	-0.155	0.013	-6.943	PPP1CC	5501	NM_002710	4506006
H08	92	-0.134	-0.128	-0.175	-0.146	0.026	-3.220	NUDT3	11165	NM_006703	37622350
B04	16	-0.109	-0.158	-0.153	-0.140	0.027	-2.950	PTPN5	84867	NM_001039970	90652860
E02	50	-0.119	-0.151	-0.111	-0.127	0.021	-3.417	PHPT1	29085	NM_014172	142373598
C03	27	-0.127	-0.129	-0.120	-0.125	0.005	-14.473	NUDT14	256281	NM_177533	37594466
C11	35	-0.126	-0.065	-0.163	-0.118	0.049	-1.349	PPP2R4	5524	NM_178003	30065648
B02	14	-0.126	-0.113	-0.086	-0.108	0.020	-3.030	SGPP1	81537	NM_030791	40254975
F06	66	-0.108	-0.101	-0.103	-0.104	0.003	-17.617	NT5C3	51251	NM_016489	70608210
G03	75	-0.084	-0.111	-0.105	-0.100	0.014	-3.961	PPFIA1	8500	NM_003626	29171751
H04	88	-0.121	-0.076	-0.100	-0.099	0.022	-2.512	DUSP3	1845	NM_004090	37655179
A08	8	-0.116	-0.106	-0.075	-0.099	0.021	-2.615	CDC25A	993	NM_201567	42490759
F03	63	-0.097	-0.115	-0.083	-0.098	0.016	-3.451	DUSP21	63904	NM_022076	33942082
H11	95	-0.128	-0.067	-0.096	-0.097	0.031	-1.794	CILP	8483	NM_003613	51944961
D03	39	-0.072	-0.144	-0.067	-0.094	0.043	-1.237	CTDP1	9150	NM_048368	67188549
C06	30	-0.081	-0.102	-0.091	-0.092	0.010	-4.989	PTPRD	5789	NM_130393	104486447
C09	33	-0.091	-0.076	-0.092	-0.086	0.009	-5.295	PSPH	5723	NM_004577	46249387
G08	80	-0.116	-0.085	-0.058	-0.086	0.029	-1.674	FLJ25449	151649	NM_144714	109627669
E10	58	-0.053	-0.117	-0.081	-0.084	0.032	-1.492	PGAM2	5224	NM_000290	50593009
H09	93	-0.100	-0.110	-0.041	-0.083	0.037	-1.259	NT5C1A	84618	NM_032526	14210537
G07	79	-0.092	-0.085	-0.069	-0.082	0.012	-3.804	PPP2R3A	5523	NM_002718	32967594
F11	71	-0.084	-0.077	-0.073	-0.078	0.006	-7.842	PTPN18	26469	NM_014369	18375654
C05	29	-0.104	-0.076	-0.053	-0.078	0.025	-1.725	DUSP5	1847	NM_004419	62865889
F08	68	-0.144	-0.036	-0.053	-0.077	0.058	-0.750	ACP2	53	NM_001610	4557009
H06	90	-0.068	-0.078	-0.062	-0.069	0.008	-4.764	PTPN6	5777	NM_080548	94557302
C04	28	-0.040	-0.083	-0.076	-0.067	0.023	-1.619	EYA4	2070	NM_172103	98991762
D06	42	-0.069	-0.082	-0.040	-0.064	0.022	-1.661	PIB5PA	27124	NM_001002837	50726959
D08	44	-0.013	-0.091	-0.085	-0.063	0.043	-0.819	PPM1B	5495	NM_001033556	75813617
D04	40	-0.047	-0.073	-0.069	-0.063	0.014	-2.506	NUDT15	55270	NM_018283	8922791
A10	10	-0.126	-0.037	-0.024	-0.062	0.056	-0.630	PFKFB1	5207	NM_002625	118582285
G06	78	-0.059	-0.034	-0.088	-0.060	0.027	-1.260	PPP3R1	5534	NM_000945	45238847
D02	38	-0.052	-0.048	-0.071	-0.057	0.012	-2.661	PTPRT	11122	NM_007050	148539859
A04	4	-0.072	-0.031	-0.055	-0.053	0.021	-1.428	PPP1R3C	5507	NM_005398	21314622
F02	62	-0.120	0.004	-0.027	-0.048	0.064	-0.419	PPM1A	5494	NM_021003	29557948
H02	86	-0.047	-0.081	-0.013	-0.047	0.034	-0.787	DUSP14	11072	NM_007026	148596940
G04	76	-0.066	-0.052	-0.021	-0.047	0.023	-1.142	PPP1R14B	26472	XM_370630	41200335
H05	89	-0.052	-0.075	-0.003	-0.043	0.037	-0.664	ILKAP	80895	NM_030768	29171685
A01	1	-0.055	-0.052	-0.013	-0.040	0.023	-0.967	Non-targeted control			
E05	53	-0.067	-0.030	0.006	-0.030	0.037	-0.465	DLG1	1739	NM_001098424	148539627
B03	15	-0.053	-0.044	0.009	-0.029	0.033	-0.492	PPP1R3A	5506	NM_002711	34304342
D01	37	-0.044	-0.006	-0.037	-0.029	0.020	-0.825	Non-targeted control			
D11	47	-0.030	-0.044	-0.004	-0.026	0.020	-0.736	NUDT1	4521	NM_002452	40288273
B09	21	-0.093	-0.001	0.018	-0.025	0.059	-0.241	G6PC3	92579	NM_138387	24308433
E07	55	-0.025	-0.035	0.000	-0.020	0.018	-0.636	MK-STYX	51657	NM_016086	32481212
C10	34	-0.017	-0.054	0.010	-0.020	0.032	-0.351	CDC25C	995	NM_022809	125625348
G05	77	-0.017	0.022	-0.060	-0.018	0.041	-0.254	PTPRA	5786	NM_080841	125987586
B08	20	-0.015	-0.017	-0.019	-0.017	0.002	-5.752	PPP1R1B	84152	NM_181505	142371393
H03	87	-0.062	0.008	0.016	-0.013	0.043	-0.168	SSH1	54434	NM_018984	40254883
C08	32	-0.011	0.003	-0.016	-0.008	0.010	-0.458	MTM1	4534	NM_000252	4557895
B10	22	-0.048	-0.020	0.047	-0.007	0.049	-0.079	FLJ32332	132160	NM_144641	21389470
C01	25	-0.012	0.002	-0.010	-0.007	0.007	-0.518	Non-targeted control			
A12	12	-0.003	-0.045	0.036	-0.004	0.040	-0.056	Non-targeted control			
A03	3	-0.029	-0.023	0.046	-0.002	0.042	-0.025	DUSP10	11221	NM_144729	21536332
D12	48	0.011	-0.002	-0.005	0.001	0.009	0.093	Non-targeted control			
B11	23	-0.027	0.010	0.029	0.004	0.029	0.076	PTPRO	5800	NM_002848	13677212
H10	94	-0.055	-0.007	0.075	0.005	0.066	0.039	DUSP19	142679	NM_080876	31377625
A02	2	-0.042	0.009	0.047	0.005	0.045	0.064	DOT1L	84444	NM_032482	163965372
D10	46	0.008	-0.014	0.030	0.008	0.022	0.213	PPP4C	5531	NM_002720	4506026
B05	17	-0.033	0.015	0.048	0.010	0.041	0.139	DUSP11	8446	NM_003584	4503414
E11	59	-0.005	0.018	0.022	0.012	0.015	0.455	EPM2A	7957	NM_001018041	66346727
B01	13	0.003	0.018	0.018	0.013	0.009	0.837	Non-targeted control			
G10	82	-0.046	0.055	0.045	0.018	0.055	0.183	INPP5E	56623	NM_019892	38327538
E03	51	-0.025	0.006	0.075	0.019	0.051	0.205	PTPN21	11099	NM_007039	150418004
C02	26	-0.021	0.036	0.048	0.021	0.037	0.324	ALPL	249	NM_000478	116734716
C12	36	0.052	0.012	0.005	0.023	0.025	0.518	Non-targeted control			
A05	5	-0.005	0.005	0.074	0.024	0.043	0.320	CTDSP1	58190	NM_021198	10864008
C07	31	0.039	0.042	-0.003	0.026	0.025	0.593	DUSP4	1846	NM_001394	58331238
A11	11	0.004	0.040	0.096	0.047	0.047	0.566	ALPPL2	251	NM_031313	157266295
B12	24	0.049	0.023	0.076	0.050	0.026	1.061	Non-targeted control			
A09	9	0.017	0.034	0.109	0.053	0.049	0.611	PPP3CA	5530	NM_000944	142369518
G11	83	0.053	0.049	0.095	0.066	0.025	1.474	KIAA0310	9919	NM_014866	124378038
A06	6	0.086	0.022	0.140	0.083	0.059	0.793	PTPRJ	5795	NM_001098503	148728159
E09	57	0.049	0.086	0.117	0.084	0.034	1.404	PTPN13	5783	NM_006264	5453991

B06	18	0.057	0.107	0.132	0.098	0.038	1.458	PTPDC1	138639	NM_152422	30089951
A07	7	0.067	0.059	0.172	0.099	0.063	0.888	NT5M	56953	NM_020201	74229030

		Difference from median of controls									
Well Name	Well #	1st	2nd	3rd	Average	Standard deviation	SSMD	GeneSymbol	GeneId	GeneAccession	GINumber
F01	61	-1.238	-1.115	-1.152	-1.168	0.063	-10.424	Dynamin-2			
E01	49	-1.233	-1.118	-1.114	-1.155	0.068	-9.633	Dynamin-2			
E12	60	-1.198	-1.076	-1.142	-1.139	0.061	-10.536	Dynamin-2			
F12	72	-1.213	-1.060	-1.124	-1.132	0.077	-8.292	Dynamin-2			
G12	84	-1.008	-0.751	-0.998	-0.919	0.146	-3.563	Clathrin heavy chain			
H12	96	-1.020	-0.714	-0.956	-0.897	0.162	-3.128	Clathrin heavy chain			
H01	85	-0.960	-0.622	-0.907	-0.830	0.181	-2.580	Clathrin heavy chain			
G01	73	-0.893	-0.652	-0.900	-0.815	0.141	-3.257	Clathrin heavy chain			
F09	69	-0.365	-0.372	-0.442	-0.393	0.042	-5.247	THTPA	79178	NM_024328	54607163
G10	82	-0.330	-0.188	-0.389	-0.302	0.103	-1.652	PPP3R2	5535	NM_147180	34303920
B05	17	-0.288	-0.184	-0.366	-0.279	0.091	-1.726	PPP1R7	5510	NM_002712	4506012
F06	66	-0.256	-0.167	-0.197	-0.207	0.045	-2.594	DKFZP761G058	152926	NM_152542	31542532
C07	31	-0.209	-0.124	-0.283	-0.205	0.079	-1.456	PPP1R2	5504	NM_006241	34147499
G04	76	-0.235	-0.154	-0.209	-0.199	0.041	-2.722	SSH3	54961	NM_017857	24586674
G02	74	-0.226	-0.148	-0.186	-0.187	0.039	-2.689	PPP2R5D	5528	NM_006245	31083266
C08	32	-0.143	-0.203	-0.195	-0.180	0.033	-3.132	WBP11	51729	NM_016312	18375679
G09	81	-0.302	-0.020	-0.190	-0.171	0.142	-0.680	PPP1R12B	4660	NM_032103	14195596
C05	29	-0.164	-0.131	-0.209	-0.168	0.039	-2.404	PPP1R1C	151242	NM_001080545	122937385
D03	39	-0.187	-0.092	-0.179	-0.153	0.052	-1.646	BPNT1	10380	NM_006085	116812594
B03	15	-0.181	-0.083	-0.178	-0.147	0.056	-1.495	PTPN14	5784	NM_005401	34328898
H06	90	-0.177	-0.067	-0.176	-0.140	0.063	-1.255	NT5E	4907	NM_002526	4505466
H08	92	-0.162	-0.108	-0.150	-0.140	0.029	-2.766	MTMR6	9107	NM_004685	134142347
A06	6	-0.188	-0.089	-0.135	-0.137	0.049	-1.573	NT5C	30833	NM_014595	7657032
D09	45	-0.136	-0.050	-0.212	-0.132	0.081	-0.922	PPP2R1B	5519	NM_181699	124269711
A09	9	-0.192	-0.064	-0.132	-0.129	0.064	-1.143	PTPN23	25930	NM_015466	110681717
B04	16	-0.123	-0.102	-0.139	-0.121	0.019	-3.632	PPP2R2B	5521	NM_181676	32307116
F04	64	-0.143	-0.099	-0.100	-0.114	0.025	-2.577	DUSP15	128853	NM_177991	29826316
C06	30	-0.098	-0.061	-0.168	-0.109	0.054	-1.133	PTP4A3	11156	NM_007079	14589853
B07	19	-0.213	-0.041	-0.068	-0.107	0.093	-0.653	KIAA0377	9677	NM_014659	7662083
D10	46	-0.102	-0.113	-0.102	-0.105	0.006	-9.176	FBP2	8789	NM_003837	22907027
G07	79	-0.035	-0.123	-0.154	-0.104	0.062	-0.948	PTPN4	5775	NM_002830	18104987
H05	89	-0.127	-0.052	-0.126	-0.102	0.043	-1.329	DUSP18	150290	NM_152511	51093844
C02	26	-0.061	-0.133	-0.107	-0.100	0.036	-1.567	PPP5C	5536	NM_006247	20127496
D04	40	-0.119	-0.073	-0.102	-0.098	0.023	-2.395	ACP5	54	NM_001611	161377449
C04	28	-0.147	-0.058	-0.084	-0.096	0.046	-1.188	PPAP2C	8612	NM_003712	29171743
E08	56	-0.230	-0.018	-0.039	-0.096	0.117	-0.463	PPP1R12A	4659	NM_002480	4505316
F05	65	-0.106	-0.087	-0.092	-0.095	0.010	-5.456	PPP1R13B	23368	NM_015316	121114286
E02	50	-0.100	-0.062	-0.120	-0.094	0.029	-1.795	PPFIA4	8497	NM_015053	55769553
F02	62	-0.117	-0.039	-0.109	-0.088	0.043	-1.161	NUDT4	11163	NM_019094	98985815
A08	8	-0.092	-0.089	-0.046	-0.075	0.026	-1.662	PPP2R1A	5518	NM_014225	32455242
C09	33	-0.071	-0.007	-0.137	-0.072	0.065	-0.625	PPP1R1A	5502	NM_006741	155030237
F07	67	-0.090	-0.060	-0.060	-0.070	0.017	-2.321	MTMR7	9108	NM_004686	142379887
E03	51	-0.093	-0.026	-0.079	-0.066	0.035	-1.061	IGBP1	3476	NM_001551	48375183
D05	41	-0.151	-0.040	-0.006	-0.065	0.076	-0.487	INPP5A	3632	NM_005539	109702905
C11	35	-0.140	0.015	-0.063	-0.063	0.078	-0.458	PTPLB	201562	NM_198402	158819030
D08	44	-0.047	-0.064	-0.067	-0.059	0.011	-3.152	PPM1F	9647	NM_014634	29826333
F03	63	-0.051	-0.038	-0.076	-0.055	0.019	-1.647	PTPN1	5770	NM_002827	18104977
H11	95	-0.059	-0.031	-0.074	-0.054	0.022	-1.407	Empty			
D06	42	-0.049	-0.017	-0.088	-0.051	0.035	-0.821	PPM1E	22843	NM_014906	30089947
E06	54	-0.050	-0.065	-0.034	-0.049	0.015	-1.816	PPP1R8	5511	NM_014110	20336237
E04	52	-0.096	-0.009	-0.030	-0.045	0.045	-0.559	DUSP13	51207	NM_016364	56237017
F08	68	-0.067	-0.039	-0.025	-0.044	0.021	-1.154	CTDSP2	10106	NM_005730	93004101
C10	34	-0.015	-0.056	-0.051	-0.041	0.022	-1.023	SGPP2	130367	NM_152386	23510288
A01	1	-0.054	-0.035	-0.031	-0.040	0.012	-1.897	Non-targeted control			
G03	75	-0.019	-0.046	-0.053	-0.039	0.018	-1.219	ACPP	55	NM_001099	161377405
A11	11	-0.058	-0.031	-0.022	-0.037	0.019	-1.107	PPAP2B	8613	NM_003713	29171739
F10	70	-0.019	-0.015	-0.077	-0.037	0.035	-0.603	DUSP22	56940	NM_020185	34147625
E05	53	-0.050	-0.007	-0.052	-0.036	0.026	-0.801	SBF1	6305	NM_002972	37574611
D11	47	-0.045	-0.025	-0.034	-0.035	0.010	-2.010	DUSP7	1849	NM_001947	85662716
D01	37	-0.027	-0.033	-0.036	-0.032	0.004	-4.233	Non-targeted control			
E09	57	-0.123	0.034	0.001	-0.030	0.083	-0.202	C9ORF67	84814	NM_032728	34147435
H04	88	-0.040	0.008	-0.054	-0.029	0.032	-0.500	PTPRG	5793	NM_002841	18860897
H03	87	-0.004	-0.004	-0.072	-0.027	0.039	-0.387	PPFIA3	8541	NM_003660	41872688
H09	93	0.011	-0.025	-0.056	-0.023	0.033	-0.392	PPM1G	5496	NM_002707	29826283
B11	23	-0.036	-0.002	-0.025	-0.021	0.017	-0.688	PPP6C	5537	NM_002721	20127429
C01	25	-0.006	-0.020	-0.033	-0.020	0.013	-0.845	Non-targeted control			
A07	7	0.008	-0.031	-0.035	-0.019	0.024	-0.456	PPM2C	54704	NM_018444	8923959
B09	21	-0.015	-0.004	-0.034	-0.017	0.016	-0.635	TENS1	64759	NM_022748	134152712
G05	77	-0.008	0.005	-0.049	-0.017	0.028	-0.345	LPPR4	9890	NM_014839	45827807
G06	78	0.013	-0.047	-0.016	-0.017	0.030	-0.315	PTPN22	26191	NM_012411	15619017
D07	43	-0.009	-0.023	-0.014	-0.015	0.007	-1.208	FBP1	2203	NM_000507	160298191
G11	83	-0.037	0.002	0.009	-0.009	0.025	-0.198	CDC14B	8555	NM_001077181	116008457
B01	13	-0.015	-0.004	-0.008	-0.009	0.006	-0.885	Non-targeted control			
H10	94	-0.042	-0.008	0.036	-0.005	0.039	-0.072	ALPP	250	NM_001632	94721245
A03	3	0.000	-0.015	0.010	-0.001	0.013	-0.063	G6PC2	57818	NM_001081686	126273545
H02	86	-0.016	-0.007	0.022	0.000	0.020	-0.006	NUDT5	11164	NM_014142	37594463
F11	71	0.040	-0.017	-0.023	0.000	0.035	0.000	PTPRR	5801	NM_002849	19743915
C03	27	0.009	0.000	-0.006	0.001	0.007	0.089	TPTE2	93492	NM_130785	40549428
G08	80	0.045	-0.008	-0.026	0.004	0.037	0.055	PPFIBP2	8495	NM_003621	57163846
E11	59	-0.050	0.058	0.023	0.011	0.055	0.109	CDC25B	994	NM_004358	47078250
E10	58	-0.044	0.042	0.068	0.022	0.059	0.210	NUDT12	83594	NM_031438	37574615
D12	48	0.006	0.041	0.027	0.025	0.017	0.798	Non-targeted control			
C12	36	0.067	0.004	0.008	0.026	0.036	0.416	Non-targeted control			
A04	4	-0.029	0.075	0.034	0.027	0.052	0.290	PFKFB2	5208	NM_001018053	64762444
A12	12	0.060	0.005	0.019	0.028	0.028	0.558	Non-targeted control			
A02	2	0.029	0.028	0.048	0.035	0.011	1.747	ACP6	51205	NM_016361	151301212
B06	18	0.036	0.041	0.052	0.043	0.008	3.099	SSH2	85464	NM_033389	37674209
B10	22	0.049	0.061	0.045	0.052	0.008	3.452	PON2	5445	NM_001018161	66529395
B08	20	0.010	0.045	0.103	0.053	0.047	0.635	LCK	3932	NM_001042771	112789547
B12	24	0.041	0.025	0.097	0.054	0.038	0.810	Non-targeted control			
A10	10	0.021	0.044	0.102	0.055	0.042	0.749	CDKN3	1033	NM_005192	17981703
E07	55	0.020	0.078	0.118	0.072	0.049	0.828	NT5C1B	93034	NM_033253	50593107
H07	91	0.084	0.054	0.109	0.082	0.027	1.689	PPM1L	151742	NM_139245	63003904
D02	38	0.081	0.064	0.117	0.087	0.027	1.809	ALPI	248	NM_001631	157266291

B02	14	0.078	0.113	0.088	0.093	0.018	2.907	IMPA2	3613	NM_014214	7657235
A05	5	0.106	0.081	0.174	0.120	0.048	1.407	PFKFB4	5210	NM_004567	19923257

		Difference from median of controls									
Well Name	Well #	1st	2nd	3rd	Average	Standard deviation	SSMD	GeneSymbol	GeneId	GeneAccession	GINumber
E01	49	-1.174	-1.149	-1.219	-1.181	0.036	-18.749	Dynamin-2			
F01	61	-1.155	-1.105	-1.230	-1.163	0.063	-10.428	Dynamin-2			
E12	60	-1.188	-1.072	-1.213	-1.158	0.075	-8.661	Dynamin-2			
F12	72	-1.130	-1.103	-1.239	-1.157	0.072	-9.083	Dynamin-2			
H12	96	-0.998	-0.730	-1.018	-0.915	0.160	-3.217	Clathrin heavy chain			
G12	84	-0.985	-0.664	-1.068	-0.906	0.214	-2.391	Clathrin heavy chain			
G01	73	-0.874	-0.671	-0.957	-0.834	0.147	-3.198	Clathrin heavy chain			
H01	85	-0.833	-0.652	-0.976	-0.820	0.162	-2.850	Clathrin heavy chain			
G10	82	-0.302	-0.360	-0.510	-0.391	0.107	-2.059	INPP5D	3635	NM_005541	64085176
E10	58	-0.337	-0.205	-0.271	-0.271	0.066	-2.315	PPP2CA	5515	NM_002715	57222566
G04	76	-0.212	-0.234	-0.365	-0.270	0.082	-1.849	PTPRE	5791	NM_006504	40805849
F03	63	-0.337	-0.143	-0.194	-0.224	0.101	-1.258	PPP1R15B	84919	NM_032833	41282220
H06	90	-0.141	-0.172	-0.260	-0.191	0.062	-1.744	PPP2R5B	5526	NM_006244	30795206
H07	91	-0.219	-0.101	-0.215	-0.178	0.067	-1.499	RNGTT	8732	NM_003800	134142827
E09	57	-0.206	-0.073	-0.241	-0.174	0.089	-1.105	INPPL1	3636	NM_001567	4755141
C11	35	-0.246	-0.008	-0.232	-0.162	0.133	-0.685	TIMM50	92609	NM_001001563	48526508
C06	30	-0.180	-0.089	-0.202	-0.157	0.060	-1.475	PPAP2A	8611	NM_003711	29171735
G09	81	-0.138	-0.125	-0.181	-0.148	0.030	-2.819	SACM1L	22908	NM_014016	7662337
F06	66	-0.117	-0.115	-0.194	-0.142	0.045	-1.781	PALD	27143	NM_014431	51100971
F11	71	-0.184	-0.043	-0.191	-0.139	0.083	-0.945	PPP3CB	5532	NM_021132	11036639
G07	79	-0.101	-0.098	-0.217	-0.139	0.068	-1.154	PSPHL	8781	XM_926720	89024914
H04	88	-0.100	-0.091	-0.214	-0.135	0.069	-1.110	NUDT2	318	NM_001161	22265329
H08	92	-0.136	-0.111	-0.139	-0.129	0.015	-4.754	PPP1CA	5499	NM_206873	45827797
F07	67	-0.076	-0.147	-0.162	-0.128	0.046	-1.563	PGAM1	5223	NM_002629	31543395
C05	29	-0.096	-0.120	-0.144	-0.120	0.024	-2.823	DUSP9	1852	NM_001395	4503420
E05	53	-0.153	-0.094	-0.108	-0.118	0.031	-2.154	PPP2R5A	5525	NM_006243	30795205
G08	80	-0.089	-0.097	-0.161	-0.116	0.040	-1.646	DUSP6	1848	NM_001946	42764682
H02	86	-0.096	-0.102	-0.144	-0.114	0.026	-2.469	PTPRK	5796	NM_002844	18860901
E07	55	-0.126	-0.063	-0.139	-0.109	0.041	-1.514	DUSP12	11266	NM_007240	6005955
B06	18	-0.106	-0.075	-0.139	-0.107	0.032	-1.878	DLG7	9787	NM_014750	21361644
C08	32	-0.137	-0.017	-0.143	-0.099	0.071	-0.790	INPP4B	8821	NM_001101669	156104896
F02	62	-0.116	-0.092	-0.082	-0.097	0.017	-3.142	PTPRB	5787	NM_002837	157952212
H11	95	-0.081	-0.059	-0.148	-0.096	0.047	-1.165	PPP4R1	9989	NM_005134	108936951
H05	89	-0.059	-0.076	-0.144	-0.093	0.045	-1.169	SKIP	51763	NM_016532	18765708
D11	47	-0.137	0.015	-0.155	-0.092	0.093	-0.560	DUSP1	1843	NM_004417	7108342
C09	33	-0.102	-0.072	-0.101	-0.092	0.017	-3.018	MTMR3	8897	NM_153051	148276975
B09	21	-0.157	-0.021	-0.092	-0.090	0.068	-0.748	PPP1CB	5500	NM_002709	4506004
E11	59	-0.132	-0.015	-0.114	-0.087	0.063	-0.784	TA-PP2C	160760	NM_139283	21281678
G03	75	-0.051	-0.075	-0.128	-0.085	0.039	-1.212	PTPRN	5798	NM_002846	18860905
G06	78	-0.055	-0.082	-0.114	-0.084	0.030	-1.604	PTPRS	5802	NM_130853	104487294
H03	87	-0.116	-0.030	-0.097	-0.081	0.045	-1.011	PPAPDC1	196051	NM_001030059	73611919
F04	64	-0.188	0.012	-0.066	-0.081	0.101	-0.452	INPP5B	3633	NM_005540	113722124
H09	93	-0.036	-0.082	-0.100	-0.073	0.033	-1.240	BPGM	669	NM_001724	40353767
G05	77	-0.052	-0.048	-0.118	-0.073	0.039	-1.050	DUSP16	80824	NM_030640	38372910
D06	42	-0.060	-0.046	-0.110	-0.072	0.034	-1.197	PPP2R5E	5529	NM_006246	31083295
H10	94	-0.052	-0.051	-0.103	-0.069	0.030	-1.314	PPFIA2	8499	NM_003625	29171754
B07	19	-0.021	-0.058	-0.125	-0.068	0.053	-0.725	PTPN20A	653129	NM_001042397	109138574
F05	65	-0.077	-0.052	-0.055	-0.061	0.014	-2.510	PTP4A1	7803	NM_003463	62865860
G02	74	-0.029	-0.047	-0.107	-0.061	0.041	-0.837	MTMR8	55613	NM_017677	21361758
E08	56	-0.061	0.008	-0.090	-0.048	0.050	-0.535	PPP3CC	5533	NM_005605	34147659
E02	50	-0.069	-0.033	-0.033	-0.045	0.021	-1.224	ACPT	93650	NM_033068	18375509
A06	6	-0.042	-0.027	-0.066	-0.045	0.020	-1.294	PTPRU	10076	NM_133178	110735403
D01	37	-0.058	0.000	-0.076	-0.044	0.040	-0.631	Non-targeted control			
F10	70	-0.093	0.004	-0.036	-0.041	0.049	-0.482	PTPRM	5797	NM_002845	157419149
D05	41	-0.039	-0.031	-0.052	-0.040	0.010	-2.183	PPP2CZ	333926	NM_005167	65506327
B08	20	-0.059	0.007	-0.059	-0.037	0.038	-0.548	SYNJ1	8867	NM_003895	44921605
B03	15	-0.006	-0.053	-0.045	-0.035	0.025	-0.788	PON1	5444	NM_000446	31880793
C01	25	-0.028	-0.017	-0.045	-0.030	0.014	-1.193	Non-targeted control			
B05	17	0.023	-0.058	-0.052	-0.029	0.045	-0.360	PPP2R2D	55844	NM_001003656	51093852
F09	69	-0.089	0.033	-0.008	-0.021	0.062	-0.195	SYNJ2	8871	NM_003898	52851404
A07	7	0.028	-0.051	-0.039	-0.021	0.043	-0.271	DUSP2	1844	NM_004418	12707563
G11	83	0.016	-0.005	-0.072	-0.020	0.046	-0.251	PTEN	5728	NM_000314	110224474
C12	36	-0.019	-0.030	-0.004	-0.018	0.013	-0.769	Non-targeted control			
A08	8	-0.035	-0.017	0.001	-0.017	0.018	-0.535	PPP1R3D	5509	NM_006242	37622904
C07	31	-0.003	-0.011	-0.034	-0.016	0.016	-0.553	PDP2	57546	NM_020786	41349496
A01	1	-0.025	-0.015	0.004	-0.012	0.015	-0.452	Non-targeted control			
E06	54	-0.044	0.036	-0.016	-0.008	0.041	-0.113	PTPMT1	114971	NM_175732	148224883
E04	52	-0.013	0.030	-0.030	-0.004	0.031	-0.074	PTPLA	9200	NM_014241	82659104
B11	23	0.077	-0.007	-0.072	-0.001	0.075	-0.005	IMPA1	3612	NM_005536	8393607
C02	26	-0.019	0.001	0.016	-0.001	0.017	-0.018	PPM1D	8493	NM_003620	29558519
B01	13	0.022	0.020	-0.031	0.004	0.030	0.068	Non-targeted control			
B02	14	0.077	-0.009	-0.045	0.008	0.063	0.070	STYX	6815	NM_145251	34222188
D09	45	-0.020	0.002	0.045	0.009	0.033	0.155	MTMR2	8898	NM_016156	20357517
E03	51	0.015	0.023	-0.003	0.012	0.013	0.509	PTPRZ1	5803	NM_002851	91208427
A11	11	0.086	-0.006	-0.035	0.015	0.063	0.136	ENPP7	339221	NM_178543	31341740
C03	27	0.031	0.010	0.007	0.016	0.013	0.682	PTPRC	5788	NM_080923	115385974
B04	16	0.044	0.001	0.024	0.023	0.021	0.613	MGC10067	134510	NM_145049	142347678
D08	44	0.060	0.025	-0.003	0.027	0.032	0.483	OCRL	4952	NM_000276	21396493
A03	3	0.010	0.052	0.022	0.028	0.022	0.724	MINPP1	9562	NM_004897	164420775
A05	5	-0.003	0.038	0.051	0.029	0.028	0.569	PTPN12	5782	NM_002835	18375651
D02	38	-0.002	0.025	0.063	0.029	0.033	0.498	EYA1	2138	NM_172059	26667218
A10	10	0.083	-0.004	0.019	0.033	0.045	0.411	PGAM4	441531	NM_001029891	84370365
D12	48	0.019	0.035	0.049	0.034	0.015	1.277	Non-targeted control			
B12	24	0.062	0.036	0.027	0.042	0.018	1.273	Non-targeted control			
A12	12	0.071	0.000	0.068	0.046	0.040	0.648	Non-targeted control			
F08	68	0.030	0.097	0.022	0.050	0.041	0.682	NUDT9	53343	NM_024047	37594462
C10	34	-0.037	0.116	0.105	0.061	0.086	0.403	ACP1	52	NM_001040649	96304456
D07	43	0.050	0.055	0.078	0.061	0.015	2.356	PPP2CB	5516	NM_004156	57634540
D04	40	0.058	0.053	0.097	0.069	0.024	1.620	MTMR1	8776	NM_003828	88999580
A09	9	0.095	0.062	0.050	0.069	0.023	1.689	PTP4A2	8073	NM_080392	88758598
A02	2	0.104	0.065	0.044	0.071	0.031	1.295	PPP1R16B	26051	NM_015568	34335276
D10	46	0.063	0.070	0.088	0.074	0.013	3.225	PPEF1	5475	NM_152226	23312377

B10	22	0.098	0.083	0.051	0.077	0.024	1.811	DUSP8	1850	NM_004420	153281157
A04	4	0.106	0.062	0.102	0.090	0.024	2.087	PTPN9	5780	NM_002833	18375663
C04	28	0.045	0.087	0.140	0.091	0.048	1.074	PPAPDC2	403313	NM_203453	66773039
D03	39	0.097	0.108	0.111	0.105	0.007	7.940	NT5C2	22978	NM_012229	20149601
